# Mobile phone use and risk of glioma in adults: case–control study

**DOI:** 10.1038/sj.bjc.6603068

**Published:** 2006-03-28

**Authors:** S Milham

**Affiliations:** 1 2318 Gravelly Beach Loop NW, Olympia, WA 98502, USA

**Sir**,

I am disturbed by the conclusion of the paper ‘Mobile phone use and the risk of acoustic neuroma: results of the Interphone case–control study in five Northern European countries, by MJ Schoemaker *et al*, *British Journal of Cancer* doi:10.1038/sj.bjc.6602764. The authors conclude that ‘…there is no substantial risk of acoustic neuroma in the first decade after starting mobile phone use.’

Tables 2 and 3 present risks (odds ratios) of acoustic neuroma in relation to various characteristics of mobile phone use. In these two tables, 36 odds ratios were presented: three were at 1.0, 28 were below 1.0, and five were above 1.0. If there is no relation between cell phone use and the development of acoustic neuroma, I would expect as many odds ratios above as below 1.0. The chance of observing either 28 or more successes or five or fewer successes in 33 binomial trials is *P*<0.0001. Interestingly, all five of the odds ratios above 1.0 were in long-term cell phone users (over 10 years) or in users with high cumulative hours of use. The only statistically significant odds ratio was seen in ipsilateral tumors in the long-term cell phone users (over 10 years). These findings suggest that either cell phone use protects against acoustic neuroma or there is a problem with exposure assessment. With a nonresponse rate of about 40% in the controls, I think that a more likely explanation for these results is that the controls who responded to the mailed invitation to participate in the study were more likely to be cell phone users than the nonparticipating controls. Given the long latencies of solid tumors in humans, I think that the pattern of these results suggests that we may be at the beginning of an epidemic of cell phone induced tumors, rather than the authors’ claim of, ‘… no substantial risk.’

