# Mobile phone use and risk of glioma in adults: case–control study

**DOI:** 10.1038/sj.bjc.6603128

**Published:** 2006-05-02

**Authors:** S Milham

**Correction to**: *British Journal of Cancer* (2006) **94**, 1351. doi:10.1038/sj.bjc.6603068

Owing to an author error, the title of the above letter was incorrect. The title should be:

‘Mobile phone use and risk of acoustic neuroma: results of the Interphone case–control study in five North European countries’.

